# SOD1 misplacing and mitochondrial dysfunction in amyotrophic lateral sclerosis pathogenesis

**DOI:** 10.3389/fncel.2015.00336

**Published:** 2015-08-25

**Authors:** Francesco Tafuri, Dario Ronchi, Francesca Magri, Giacomo P. Comi, Stefania Corti

**Affiliations:** Dino Ferrari Centre, Neuroscience Section, Department of Pathophysiology and Transplantation (DEPT), University of Milan, Neurology Unit, IRCCS Foundation Ca’ Granda Ospedale Maggiore PoliclinicoMilan, Italy

**Keywords:** superoxide dismutase 1, motor neuron disorder, mitochondria, mitochondrial intermembrane space, outer mitochondrial membrane, protein misfolding

## Abstract

Amyotrophic lateral sclerosis (ALS) is a fatal motor neuron disease presenting as sporadic (sALS) or familial (fALS) forms. Even if the list of the genes underlining ALS greatly expanded, defects in superoxide dismutase 1 (*SOD1*), encoding the copper/zinc SOD1, still remain a major cause of fALS and are likely involved also in apparently sporadic presentations. The pathogenesis of ALS is still unknown, but several lines of evidence indicate that the mitochondrial accumulation of mutant SOD1 is an important mechanism of mitochondrial dysfunction, leading to motor neuron pathology and death. The intramitochondrial localization of mutant SOD1 is debated. Mutant SOD1 might accumulate inside the intermembrane space (IMS), overriding the physiological retention regulated by the copper chaperone for superoxide dismutase (CCS). On the other hand, misfolded SOD1 might deposit onto the outer mitochondrial membrane (OMM), clumping the transport across mitochondrial membranes and engaging mitochondrial-dependent cell apoptosis. The elucidation of the mechanisms ruling SOD1 localization and misplacing might shed light on peculiar ALS features such as cell selectivity and late onset. More importantly, these studies might disclose novel targets for therapeutic intervention in familial ALS as well as non-genetic forms. Finally, pharmacological or genetic manipulation aimed to prevent or counteract the intracellular shifting of mutant SOD1 could be effective for other neurodegenerative disorders featuring the toxic accumulation of misfolded proteins.

## Introduction

Amyotrophic lateral sclerosis (ALS) is a lethal neurological disease caused by the selective degeneration of upper and lower motor neurons, resulting in progressive muscle denervation and paralysis (Bucchia et al., 2015). Recent studies also feature non-cell autonomous mechanisms involving contiguous and functionally related cells such as astrocytes, oligodendrocytes, microglia as well as the extracellular environment (Rizzo et al., 2014). No effective therapy is available for ALS and understanding the disease pathogenesis could help in developing effective treatments (Bucchia et al., 2015).

The majority of ALS cases are sporadic (sALS) while familial (fALS) forms account for 10% of all ALS presentations (Renton et al., 2014). Nevertheless, sALS and fALS share similar phenotypic features, being clinically indistinguishable, with fALS patients only displaying, sometimes, earlier onset respect to sporadic probands. To date, the genetic defect underlining ALS is identified in approximately two thirds of fALS cases, while nine of ten sporadic forms lack a molecular diagnosis (Renton et al., 2014). The application of massive parallel sequencing techniques to undiagnosed cases is expected to improve the diagnostic yield and the identification of novel genes in fALS. It is more difficult to predict if sporadic forms result from undisclosed (rare) molecular defects or are the consequence of additional factors such as environment and ageing. Indeed, the existence of multifactorial causes underlining sALS is a rapidly emerging hypothesis (Robberecht and Philips, 2013). More than 30 genes have been found mutated in ALS patients but few of them are epidemiologically relevant and currently considered for genetic testing (Renton et al., 2014). They include: *SOD1*, encoding for the copper/zinc superoxide dismutase 1, TAR DNA binding protein (*TARDBP*), fused RNA binding protein (*FUS*) and *C9orf72*, containing a pathological hexanucleotide repeat expansion which constitutes the most frequent molecular cause of sALS/fALS, towering over all other defects so far described (Renton et al., 2014).

Despite considerable efforts, the pathogenesis of ALS is still obscure. Multiple intracellular pathways have been proposed as relevant: the regulation of RNA transcription and editing, protein modification, folding and clearance, axonal transport, organelles maintenance, cell death mechanisms (Robberecht and Philips, 2013; Bucchia et al., 2015; Cozzolino et al., 2015).

The co-existence of multiple abnormalities and the plethora of results, sometimes contradictory, used to support or refuse pathogenetic hypothesis give rise to an apparently “chaotic” scenario, reflecting our limited comprehension of how the identified molecular defects result in motor neuron pathology and clinical onset. This confusing background is not surprising since many of the genes involved in ALS encode for proteins displaying multiple functions. The intracellular localization of the mutated proteins might also influence general protein expression and turnover in a cell-specific manner. In this regard, ALS has been considered a polygenic and multifactorial disorder, where derangement of multiple pathways might produce similar phenotypes sharing clinical, instrumental, and prognostic features (Robberecht and Philips, 2013). Since fALS and sALS also share common defects and similar alterations have been found in ALS animal models and patients’ tissues, the existence of conserved mechanisms underlining motor neuron pathology has been proposed.

*SOD1* mutations have been the first molecular defects described in fALS forms with dominant inheritance (Rosen et al., 1993) and most of the research studies addressing ALS pathogenesis and the development of therapeutic strategies have been performed using SOD1 transgenic models (Gurney et al., 1994). Recent discoveries in ALS genetics acknowledge the role of proteins involved in RNA metabolism and cytoskeletal organization, often enriched in central nervous tissues. Conversely, the expression of *SOD1* is ubiquitous and not restricted to nor increased in spinal cord and motor neurons. Further, SOD1 levels are not developmental-regulated. SOD1 enzyme shows a single, well-defined catalytic function, dealing with the detoxification of the superoxide specie inside cells. In the hunt for the ALS culprit, the loss of SOD1 enzymatic activity has been rapidly ruled out, since mutations preserving or abolishing SOD1 activity were found to cause ALS (Cozzolino et al., 2015). These elements alone indicate that the involvement of SOD1 in an adult onset motor neuron neurodegenerative disorder is not obvious.

The seminal observation of the co-localization of SOD1 with vacuolated mitochondria in a mouse model expressing human mutant SOD1 (Jaarsma et al., 2001) advanced the hypothesis that *SOD1* mutations might be toxic for mitochondria. These studies have been replicated and expanded in several experiments performed in tissues and cells obtained from ALS animal models and patients (Palomo and Manfredi, 2015). Mitochondrial impairment has been observed in major neurodegenerative disorders (Schon and Przedborski, 2011). Although motor neurons are highly reliant on oxidative phosphorylation, the relevance of mitochondria in ALS pathogenesis has been under evaluated for many years. However, signs of mitochondrial dysfunction have been observed in multiple ALS patients (Cozzolino et al., 2015) and tissues, including muscle (Corti et al., 2009). In a review of a large series of muscle biopsies, we found that respiratory chain impairment is a common feature of ALS muscles, sometimes preceding the motor neuron pathology (Crugnola et al., 2010), consistent with experimental models (Luo et al., 2013).

Differently from Parkinson’s disease (PD), where several genes involved in familial forms encode for mitochondrial proteins (De Rosa et al., 2015), the mitochondrial localization of products encoded by ALS-related genes is not exclusive. We previously described an out-of frame mutation in the gene encoding for a mitochondrial subunit of cytochrome c oxidase (COX) in a patient with severe muscle COX deficiency and a clinical phenotype resembling ALS (Comi et al., 1998). Similarly, primary mitochondrial disorders might present as motor neuron phenotypes (Ronchi et al., 2012). Recently, a missense mutation in *CHCHD10*, encoding for an intermembrane space (IMS) protein likely involved in mitochondrial cristae remodelling, was found to segregate with disease in a large French family displaying ALS with frontotemporal dementia (Bannwarth et al., 2014). Although additional *CHCHD10* mutations were detected in patients with motor neuron disorders (Cozzolino et al., 2015), mitochondrial dysfunction has been only occasionally documented (Ronchi et al., 2015).

**Figure 1:**
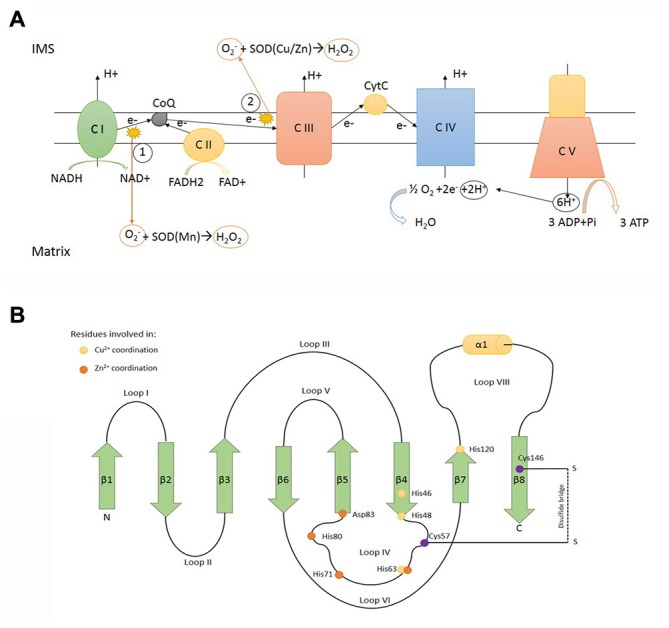
**SOD1 function and structure. (A)** Electron transport chain locatedin the inner mitochondrial membrane (IMM). (1) and (2) represent the main sources of superoxide anion (O_2_^−^), a reactive oxygen specie converted to H_2_O_2_ in a dismutase reaction catalyzed by SOD (Cu/Zn; SOD1) and SOD (Mn; SOD2) in the intermembrane mitochondrial space (IMS) and matrix, respectively. C I: complex I NADH dehydrogenase. C II: complex II succinate dehydrogenase. C III: complex III ubiquinol cytochrome C oxidoreductase. C IV: complex IV cytochrome C oxidase. C V: complex V ATP synthase. **(B)** Scheme representing the determinants of tertiary structure of human SOD1, including β sheets (green arrows), α helix (yellow cilinder) and loops. β sheets: β1: 2–8° aa -β2: 15–22° aa -β3: 29–36° aa -β4: 41–48° aa -β5: 83–89° aa -β6: 95–101° aa -β7: 116–120° aa -β8: 143–151° aa. α helix: 134–137° aa. The yellow dots represent residues involved in Cu^2+^ coordination (His46, His48, His63, His120). The orange dots represent residues involved in Zn^2+^ coordination (His63, His71, His80, Asp83). The purple dots represent residues involved in disulfide bridge formation (Cys57, Cys146).

Physiologically, a small proportion of SOD1 protein is located in the mitochondrial IMS of yeast (Sturtz et al., 2001) and mammals (Okado-Matsumoto and Fridovich, 2001; Figure 1A). A protective antioxidant role for mitochondrial SOD1 has been hypothesized (Okado-Matsumoto and Fridovich, 2001) although without conclusive evidences. Conversely, whether SOD1 mutants accumulate inside mitochondria and the consequence of such accumulation have been largely investigated. Although aberrant SOD1 was observed within mitochondrial matrix (Vijayvergiya et al., 2005), two main submitochondrial localizations for mutant SOD1 are generally acknowledged: the IMS and the outer mitochondrial membrane (OMM). In this review, we focus on the pathogenetic role of mutant SOD1 elicited by its misplacing in mitochondria, depicting the pathways ruling its targeting towards IMS or OMM. We will also comment on how these events relate to ALS pathogenesis, including cell selectivity and age of onset.

## Mitochondrial Localization of Non-Mutated SOD1

Human* SOD1* maps to chromosomal location 21q22.11 and encodes for a 17 kDa protein (Figure 1B). The encoded product, SOD1, is an enzyme which catalyses the removal of superoxide according the reaction O_2_^−^ + 2H^+^ → H_2_O_2_ (superoxide dismutation). Human genome encodes for two additional superoxide dismutases: SOD2, located inside mitochondrial matrix and the extracellular SOD3. There is no apparent evolutionary relationship between SOD1 and its isoenzymes. SOD1 is generally acknowledged as a cytosolic enzyme (Vehviläinen et al., 2014), but it is now accepted that a minor fraction (less than 5%) of total SOD1 can be found in the IMS, in lower (Sturtz et al., 2001) and higher eukaryotes (Okado-Matsumoto and Fridovich, 2001).

Mitochondrial respiratory chain, assembled inside the inner mitochondrial membrane (IMM), produces high amounts of reactive oxygen species (ROS) as a bypass product of the electron transfer reactions occurring in respiratory chain complexes (mainly complex I and complex III; Murphy, 2009). The release of superoxide anion on both sides of the inner membrane requires dedicated detoxification enzymes inside mitochondrial matrix (SOD2) and IMS (SOD1). Reactions catalysed by SOD enzymes result in the production of H_2_O_2_, another dangerous ROS able to react with Cytochrome C (Fe3+) producing the highly reactive species Oxoferryl-CytC CytC (Fe4+; Vehviläinen et al., 2014). Therefore H_2_O_2_ must be carefully neutralized and cells express catalase, glutathione peroxidase and peroxiredoxins to process H_2_O_2_ (Palomo and Manfredi, 2015). It has been proposed that, under mitochondrial stress, exceeding SOD1 activity might paradoxically boost the production of toxic ROS inside IMS by increasing H_2_O_2_ production and consequently the deleterious cytochrome-c mediated peroxidation (Goldsteins et al., 2008).

**Figure 2:**
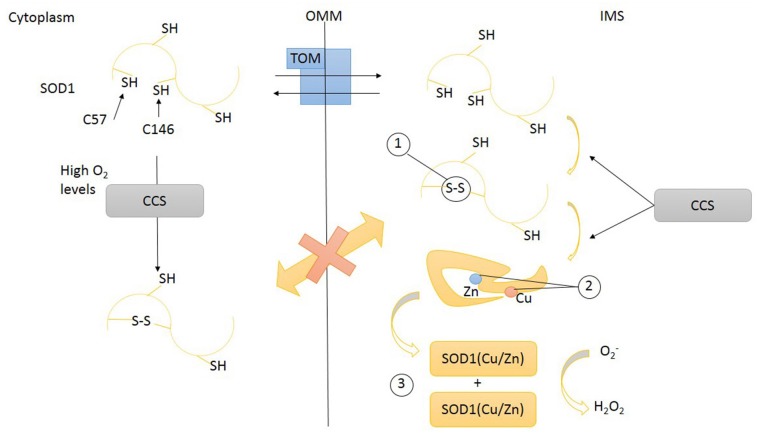
**Transport of SOD1 across mitochondrial intermembrane space (IMS).** SOD1 (unfolded) apo-protein enters IMS through the transporter of outer membrane (TOM). Inside IMS unfolded SOD1 undergoes maturation steps: (1) disulfide bonds promoting a proper folding, (2) the insertion of metal ions Zn and Cu, (3) dimerization. Disulfide bonds formation and prosthetic group insertion are mediated by copper chaperone for superoxide dismutase (CCS). Disulfide bonds prevent SOD1 leakage from mitochondria through TOM. High O_2_ levels might also induce CCS and SOD1 protein folding in cytoplasm. SOD1, superoxide dismutase 1; CCS, copper chaperone for superoxide dismutase; TOM, translocase of outer membrane; OMM, outer mitochondrial membrane; IMS, intermembrane space.

**Figure 3:**
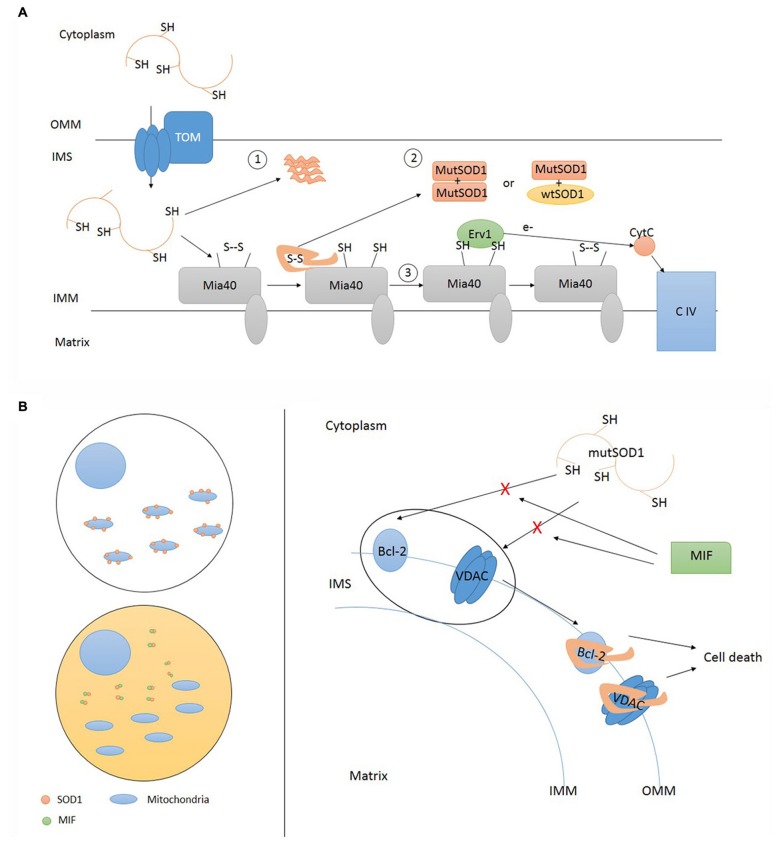
**Accumulation of mutant SOD1 inside IMS and deposition onto OMM. (A)** Mutant SOD1 enters IMS through TOM. Inside IMS mutant SOD1 can precipitate in insoluble form (1) or undergo folding via Mia40 resulting in the establishment of funtional homodimers and heterodimers (2). Mia40 transfers electrons to Cytochrome C (CytC) via Erv1 (3). TOM, translocase of outer membrane; OMM, outer mitochondrial membrane; IMS, intermembrane space; IMM, inner mitochondrial membrane; CIV, complex IV cytochrome C oxidase; MutSOD1, mutant SOD1. **(B)** Misfolded SOD1 localization upon mitochondria can be regulated in different tissue cells. Cytosol composition may differ according cell types. High cytosolic levels of soluble MIF (macrophage migration inhibitory factor) are able to prevent SOD1 mislocalization where its absence favor SOD1 deposition onto OMM. Misfolded SOD1 interactions with OMM proteins, such as Bcl-2 and VDAC, activate pro-apoptotic mitochondrial pathway leading to cell death. This interaction can be prevented by MIF. TOM, translocase of outer membrane; OMM, outer mitochondrial membrane; IMS, intermembrane space; IMM, inner mitochondrial membrane; CIV, complex IV cytochrome C oxidase; MutSOD1, mutant SOD1.

To work properly, SOD1 requires post-translational modifications including the formation of intramolecular disulfide bonds to achieve a correct folding, the binding of zinc and copper metal ions, and the exposition of a hydrophobic region required for the organization of SOD1 monomers in dimers. These post-translational changes not only are necessary for SOD1 function but also influence its subcellular localization (Figure 2). Despite the lack of a mitochondrial localization signal, after cytosolic translation, a small amount of SOD1 might enter mitochondria in an unfolded state, taking advantage of the OMM translocator TOM (Kawamata and Manfredi, 2008; Figure 3A). The copper chaperone for superoxide dismutase (CCS) promotes the establishment of disulfide bonds and catalyses the insertion of copper metal into SOD1 apoenzyme (Culotta et al., 1997). After reaching its mature form, SOD1 also remains inside IMS, with coordinated metals and disulfide bonds now preventing the leakage back to the cytosol (Kawamata and Manfredi, 2008). CCS greatly contributes to SOD1 entrapment inside IMS through the establishment of a disulfide bond between cysteines C57 and C146 and favoring the copper insertion into the apoenzyme (Kawamata and Manfredi, 2008).

CCS has been also postulated to act as a redox sensor for the subcellular distribution of SOD1. High cytosolic oxygen concentration maintains CCS in the cytosol where it folds and helps apoenzyme SOD1 to achieve a mature form. Low oxygen concentrations (hypoxia) promote the translocation of CCS across the mitochondrial membrane. Inside IMS, CCS induces the oxidative (disulfide bonds) folding of SOD1 apoezyme and inserts the copper. The rationale for this mechanism is that the expected boost of mitochondrial respiratory chain activity, induced by hypoxia, will increase the concentration of superoxide anion inside mitochondria: the enhanced translocation of CCS, promoting SOD1 maturation, might be part of a compensatory antioxidant strategy (Kawamata and Manfredi, 2008). Therefore the intracellular distribution (and activation) of SOD1 is intimately related to the intracellular location of its chaperone CCS. CCS also lacks a canonical mitochondrial localization signal and relies for its mitochondrial import on the so-called disulfide relay system (DRS), classically used by small cysteine-rich IMS proteins (Mesecke et al., 2005). This mechanism, identified in yeast 10 years ago (Mesecke et al., 2005), is quite conserved across evolution (Fischer and Riemer, 2013). The IMS proteins MIA40 and ERV1 are key players of this folding trap mechanism. When a substrate of the DRS, like apo-CCS, enters the IMS, it undergoes a MIA40-mediated oxidation involving the shifting of disulfide bonds between specific cysteine pairs (disulfide relay). This oxidation-mediated folding retains the DRS substrate inside IMS (Herrmann and Riemer, 2010). MIA40, now reduced, is re-oxidized by ERV1/ALR, a FAD-dependent sulfhydryl oxidase that transfers electrons to cytochrome C (Herrmann and Riemer, 2010). Therefore, respiratory chain is connected with the DRS, influencing the import of several mitochondrial proteins, including assembly factors involved in the assembly of respiratory chain and the maturation of inner membrane.

Several authors have demonstrated that CCS is a substrate of DRS, differently from SOD1 (Groß et al., 2011; Klöppel et al., 2011). However, a recent observation proposed that MIA40 is dispensable for oxidation-dependent import of CCS into mitochondria: CCS might directly promote its own oxidation, as well as the oxidation of SOD1 and other, still uncharacterised, substrates (Suzuki et al., 2013). These findings reinforce the link between SOD1 and its chaperone CCS in terms of activity and subcellular distribution. Interestingly, an alternative pathway for SOD1 mitochondrial translocation has been depicted in yeast, acknowledging the role of mitochondrial inner membrane organizing system (MINOS; Varabyova et al., 2013). The MINOS machinery is a recently identified protein complex involved in the organization of the IMM (Varabyova et al., 2013). Notably, CHCHD10 may be part of this complex, given its apparent function and structural similarities with other members of the MINOS system. Although the underlining mechanism is still unclear, the mitochondrial import of SOD1 is reduced in yeast mutants lacking MINOS core components. MIA40 is also fundamental for this alternative import pathway with a different role respect to that of oxidation scaffold described in the DRS (Varabyova et al., 2013). The dysfunction of DRS pathway and MINOS machinery result in clinical phenotypes with mitochondrial impairment as observed in patients harbouring mutations in core components (Di Fonzo et al., 2009) or substrates (Bannwarth et al., 2014) of these mechanisms.

## Accumulation of Mutant SOD1 in the Mitochondrial Intermembrane Space (IMS)

More than 150 *SOD1* mutations have been associated with ALS, the large part being point mutations leading to amino acid substitutions (Synofzik et al., 2012). *SOD1* mutations are dominantly inherited although recessive inheritance has been also described, especially for those variants that show low penetrance.

Accumulation, misfolding, aggregation, and precipitation of proteins seem to be common hallmark of neurodegenerative disorders (Taylor et al., 2002). SOD1 mutants so far analysed show different properties on the basis of the residues affected. As an example, several SOD1 mutants display no catalytic activity while dismutase activity is totally preserved in G93A, likely the most investigated of the ALS mutations. The conservation of catalytic activity seems also to reflect the conservation of a proper folding, assayed, before the advent of conformation specific antibodies (Rotunno and Bosco, 2013), by resistance to proteinase K digestion.

Different mutations might also show different propensities to accumulate or to aggregate in high molecular weight complexes. Presence of aggregates of mutated proteins in motor neurons is increasingly considered as a hallmark of ALS. Furthermore, a relationship between insoluble mutant SOD1 aggregates and expression of signs/symptoms has been demonstrated in many different rodent models (Johnston et al., 2000; Wang et al., 2002, 2003) in line with human findings (Kato, 2008). Despite considerable study, the mechanism that confers to SOD1 aggregates cell toxicity is still undefined. It has been hypothesized that mutant SOD1 soluble species are responsible for the disease onset (Zetterström et al., 2007; Karch et al., 2009) being, indeed, more toxic than insoluble aggregated SOD1 forms (Zetterström et al., 2007; Brotherton et al., 2012; Weichert et al., 2014). The intriguing problem of SOD1 aggregation is further complicated by the fact that experimental studies have been largely performed in lines of transgenic animals (or motor neuronal cells) with strong, although heterogeneous, overexpression of mutant proteins required to display pathological phenotype. The lack of physiological SOD1 expression and the underestimated expression of wild type alleles (normally present in almost all ALS patients) challenge the results obtained in these studies. The replication of these experiments in a patient-specific context might overcome these limitations.

Other manifestations of SOD1 mutations are protein misfolding and intracellular mislocalization. Soluble misfolded mutant SOD1 was found augmented in the mitochondria of the ALS spinal cord rodents (Zetterström et al., 2007), and the specific conformation of these forms could enhance their mitochondrial entrance. Antibodies that selectively recognize misfolded/non-native SOD1 have pointed out the link between misfolded SOD1 and mitochondria in the spinal cord of ALS rodents expressing human mutant SOD1 (Vande Velde et al., 2008; Brotherton et al., 2012). SOD1 misfolding seems to be related to different post translational mechanisms, involving disulfide bridge formation (Karch et al., 2009; Kawamata and Manfredi, 2010), incorrect C6 and C111 bond (Niwa et al., 2007) and loss of metal ion binding (Banci et al., 2008). These abnormal functions lead to altered SOD1 dimerization and to a biochemically instable tertiary structure (Banci et al., 2008, 2012). Several SOD1 mutants show instability *in vitro* (Rodriguez et al., 2002, 2005; Lindberg et al., 2005) or are found in a partially unfolded oligomerized status in mitochondria (Deng et al., 2006; Ferri et al., 2006). Remarkably, Synofzik et al. (2012) described a family showing slowly progressive ALS phenotype due to the L117V SOD1 mutation, which is similar to wild type SOD1 respect to stability and dismutase activity. These data are in line with previous findings reporting that even minor quantities of misfolded SOD1 are sufficient to prompt ALS (Jonsson et al., 2006). Indeed, overexpressed wild type SOD1 is capable to achieve an unstable (misfolded) state, causing motor neuron disease phenotype in rodents (Graffmo et al., 2013).

ALS-linked *SOD1* mutations might also affect mitochondrial localization, facilitating the crossing of the OMM. Indeed, demetalated or unfolded SOD1 enzymes due to inefficient cytosolic maturation caused by ALS mutations might increase the fraction of intramitochondrial SOD1 (Field et al., 2003). Similarly, increased mitochondrial accumulation of SOD1 (both wild type and mutant) was observed *in vitro* after experimental copper depletion (Arciello et al., 2011).

The accumulation of SOD1 in mitochondria of motor neurons has been proposed as a possible explanation for the selective motor neuron degeneration occurring in ALS (Bruijn et al., 2004). Indeed, several groups have reported that mutant SOD1 co-localizes with mitochondria by immunocytochemical and biochemical studies. Although not exclusive, brain, spinal cord and motor neurons seem to be particularly exposed to mutant SOD1 accumulation and sensitive to the resulting mitochondrial dysfunction (Jaarsma et al., 2001; Higgins et al., 2002; Mattiazzi et al., 2002; Sasaki et al., 2004; Ahtoniemi et al., 2008). Notably in most of the experiments, the accumulation of SOD1 inside mitochondria paralleled the disease progression.

Forcing the expression of constructs containing wild type and mutant *SOD1* in different cellular compartments of a mouse neuroblastoma cell line resulted in increased cell death and mitochondrial dysfunction only when mutant species were targeted to mitochondria (Takeuchi et al., 2002). Specifically, the accumulation of SOD1 in IMS seems toxic. In cultured motor neuronal cells, obligate SOD1 expression in IMS leads to mitochondrial toxicity and cell death, giving results not dissimilar to those collected expressing untargeted SOD1 mutants (Cozzolino et al., 2009; Magrané et al., 2009). Although the mitochondrial environment induced abnormal oligomerization of wild type SOD1, mutant SOD1 tends to form insoluble aggregates inside mitochondria, leading to alterations in mitochondrial morphology, function and engagement of apoptosis. The removal of the cysteines involved in disulphide bond by genetic manipulation counteracted these changes (Cozzolino et al., 2009).

A mouse line expressing G93A SOD1 mutant targeted to IMS displayed neurological deficits although less evident compared to classical G93A mouse model (lacking mitochondrial targeting but presenting a stronger expression of the transgene; Igoudjil et al., 2011). Interestingly, the selective targeting of wild type SOD1 in IMS of the knockout SOD1 mouse prevented the development of motor neuropathy and preserved axonal maintenance. The rescue effect was associated with reduced oxidative stress inside mitochondrial compartment (Fischer et al., 2011).

The groups of Jeffrey Elliot and Giovanni Manfredi authored several articles about the aberrant accumulation of mutant SOD1 inside mitochondrial IMS. First, Elliot’s team observed that G93A mice developed accelerated neurological deficits when crossed with lines overexpressing CCS in CNS tissues. The double transgenic also displayed a striking reduction of age at onset and lifespan. Protein studies revealed a significantly increased of mitochondrial SOD1 content. Consistently, the dual transgenic mice expressing wild type SOD1 did not show any abnormalities with the exception of a modest increase of mitochondrial SOD1 import. Mouse overexpressing CCS displayed a normal phenotype, rejecting the hypothesis that an overloaded DRS was at the basis of the observed mitochondrial dysfunction. Notably the genetic ablation of CCS did not prevent motor neuron phenotype and mitochondrial accumulation of mutant SOD, supporting the existence of alternative pathways for mitochondrial import of SOD1 (Son et al., 2007). The impressive phenotypic effect of CCS overexpression in presence of G93A SOD1 does not seem related to SOD1 misfolding or aggregation. The G93A substitution makes SOD1 a less adequate substrate for CCS, preserving the physical interaction with the chaperone but impairing proper oxidative folding and metallation (Proescher et al., 2008). Notably, the levels of COX activity and assembly as well as protein levels of COX-subunits were selectively reduced in spinal cord from transgenic mice, even at a pre-symptomatic stage (Son et al., 2008) likely reflecting a disturbance in COX maturation. This finding demonstrates that the abnormal mitochondrial translocation of mutant SOD1 might affect respiratory chain, hindering mitochondria from inside. However, the same authors demonstrated that the functional and biochemical defects caused by CCS overexpression depend upon the redox state of the SOD1 mutant: while G37R paralleled the findings of G93A mutation, other substitutions (G86R and L126Z) did not (Son et al., 2009). These results had been partially anticipated by *in vitro* studies of Ferri et al. (2006) who had observed a shifting of the redox potential of several SOD1 mutants inside mitochondria. Interestingly, the manipulation of redox state of mitochondrial environment by overexpression of antioxidant enzymes glutaredoxin 1 and 2 improved SOD1 solubility and reduced mitochondrial anomalous localization (Cozzolino et al., 2008; Ferri et al., 2010). In particular, increased activity of glutaredoxin 2 inside mitochondrial matrix prevented the IMS aggregation of insoluble mutant SOD1 (Ferri et al., 2010). The functional rescue of mitochondria is likely mediated by a general antioxidant pro-survival effect of glutaredoxin 2 not requiring a direct interaction with mutant SOD1: indeed, glutaredoxin 2 and SOD1 show a different intramitochondrial segregation.

Kawamata and Manfredi (2008) used mammalian cells to confirm that SOD1 subcellular distribution is driven by CCS *in vitro*. They also highlighted the importance of cysteine residues in the CCS-mediated oxidation of mutant and wild type SOD1. Finally, they found that toxic properties of mutant SOD1, including propensity to aggregate and misfolding, could override CCS-dependent mitochondrial recruitment. Once inside IMS, mutant SOD1 can preserve its soluble form and build up homo and heterodimers with wild type SOD1 or aggregate with other mutant proteins and precipitate in insoluble form. According to SOD1 mutation, one way will prevail on the other; nevertheless, these aspects are not predictive of clinical phenotype (Vehviläinen et al., 2014). The untargeted overexpression of CCS in motor neuron-like cells improved the solubility of cytosolic mutant SOD1 and increased the formation of insoluble aggregates of G93A SOD1 targeted to mitochondrial compartment (Cozzolino et al., 2009). The mitochondrial import of mutant SOD1 was enhanced by exposing NSC34 cells to inflammatory cytokines, supporting the existence of not-cell autonomous factors able to influence SOD1 localization (Ferri et al., 2008). Therefore, mutant SOD1 may escape physiological control of mitochondrial localization, performed by CCS, and remain inside mitochondria exerting toxic effects. CCS translocation into mitochondria might help to entrap mutant or wild type SOD1 enzymes inside IMS, without any influence on their transport across the OMM.

## Deposition of Mutant SOD1 Onto the Cytoplasmic Layer of Outer Mitochondrial Membrane (OMM)

The results so far presented demonstrate that both wild type and mutant SOD1 can be recovered by mitochondrial fractions of ALS animal and cell models. It is likely that the final submitochondrial location of SOD1 is the IMS and that different pathways (dependent or not on CCS) might influence this subcellular repartition. Moreover, it is conceivable that mutant SOD1 can aggregate into the IMS, as in the cytosol, contributing to mitochondrial dysfunction. Interestingly, mutant SOD1 aggregation was also observed in the mitochondrial matrix of two transgenic mice lines and the recovered SOD1 resulted less active and folded respect to cytosolic counterpart (Vijayvergiya et al., 2005). However, mitochondrial matrix has not been further investigated as a target for mutant SOD1 toxicity in other ALS models.

While both mutant and wild type enzymes have been observed inside mitochondria, the OMM has been proposed to be a more selective target for mutant SOD1 only, explaining crucial aspects of ALS pathology such as cell selectivity and symptoms onset. Two research groups have mainly contributed to this topic. Pasinelli et al. (2004) used immunoprecipitation studies to demonstrate that SOD1 associates with the OMM protein Bcl-2 in the mitochondrial fractions obtained from cells expressing wild type and mutant SOD1. These findings were also confirmed *in vivo*. Wild type and mutant SOD interacted with different domains of Bcl-2 protein. Indeed, the authors showed that mutant SOD1 alters the physiological SOD1/Bcl-2 binding and primes the formation of high molecular weight complexes containing SOD1 and Bcl-2. Interestingly, experiments performed on G93A mice and autoptic samples from a patient harbouring the A4V SOD1 mutation showed that Bcl-2 is selectively sequestered by mutant SOD1 in mitochondria isolated from spinal cord, but not liver (Pasinelli et al., 2004). In a back-to-back paper, Don Cleveland’s team supported these findings. Mutant SOD1 was preferentially recovered in the mitochondrial fraction purified by gradient centrifugation of spinal cord mitochondria from symptomatic animal models expressing mutant (G93A) but not wild type SOD1 (Liu et al., 2004). Mutant SOD1 was absent in mitochondrial preparations obtained from other tissues. The effect of CCS on mitochondrial distribution was ruled out by genetic ablation of this gene. The preferential association of mutant SOD1 with spinal cord mitochondria was also confirmed in autoptic samples, taking advantage of a selective antibody developed to recognize the epitope created by a *SOD1* truncating mutation. In a second series of experiments, the authors isolated floating mitochondria, excluding the chance of co-precipitation or co-sedimentation of interfering SOD1 located inside cytosol or other organelles (Vande Velde et al., 2008). Again, they observed that SOD1 selectively tightly associate with mitochondria from spinal cord (not the brain) in transgenic mice in an age-dependent manner. Using alkali-based extraction protocol they investigate the nature of this association. Exploiting an antibody developed to decorate only the misfolded fraction of SOD1, they conclusively observe that the mutant SOD1 associated with mitochondria is in its misfolded state, while the remaining folded protein contribute to cytosolic or mitochondrial (IMS) SOD1 content. They proposed the voltage-dependent anion channel (VDAC) channel (mitochondrial porin) as the candidate docking protein for misfolded SOD1 deposition onto OMM. VDAC was found to co-precipitate only with misfolded SOD1. Symmetrically, mutant SOD1 deposited on VDAC only when inserted in the cytosolic face of a membrane reconstructed *in vitro* (Israelson et al., 2010).

The experiments discussed so far supports the selective targeting of spinal cord mitochondria and the existence of an interaction between SOD1 and OMM proteins (Figure 3B). However, the specific target of SOD1 onto OMM (Bcl-2 vs. VDAC1) and the SOD1 specie involved in this interaction (mutant only or wild type and mutant) differed according the two groups of investigators and were object of further studies.

Bcl-2 is an integral OMM protein, while VDAC1 is a mitochondrial voltage-dependent ion channel: both are involved in the intrinsic apoptotic process. Bcl-2 regulates the release of cytochrome c by the mitochondrial channel VDAC (Shimizu et al., 1999) and inhibits proapoptotic Bax signaling. Bcl-2 has been proposed as a direct mediator of mitochondrial toxicity of mutant SOD1 since it is required to induce cytochrome c release and change in mitochondrial morphology upon exposure to mutant, but not wild type, SOD1. Notably, this effect was only observed if Bcl-2 was tethered to OMM (Pedrini et al., 2010). The authors demonstrated *in vitro* and *in vivo* that mutant SOD1 alters Bcl-2 conformation, exposing its BH3 (death) domain, without which the toxic effect was ineffective.

VDAC1 acts as a gate for ions, metabolites, nucleotides, in two different dynamic conformation states. While open it allows anions, ATP and succinate flow from mitochondria to cytoplasm; when VDAC1 is closed it acts as a small cations channel. The impairment in VDAC1 function has been also linked with mitochondrial dysfunction since the drop of ATP/ADP ratio and membrane potential drives the increase of ROS production, hence contributing to extend the amount of misfolded SOD1 (Vande Velde et al., 2008; Israelson et al., 2010). Mass spectrometry analysis revealed more widespread changes in mitochondrial protein composition and significantly reduced mitochondrial import (Li et al., 2010).

We must observe that mitochondria from different tissues have different protein repertoires. This is true for OMM (Mootha et al., 2003; Bailey et al., 2007), which is also exposed to different cytoplasm compositions (Boillée et al., 2006; Vande Velde et al., 2008; Israelson et al., 2015). Tissue type, epigenetics regulation, gene transcription, oxygen concentration and ROS production may contribute to the specification of proteins recruited onto OMM (Israelson et al., 2010). Even considering these aspects, it is difficult to think at VDAC as a selective target for mitochondrial deposition of mutant SOD1 in spinal cord motor neurons, since it is widely expressed. Moreover, protein trafficking across OMM is partially rescued by other members of the VDAC family including VDAC2 and VDAC3. The hypothesis of a direct interaction between mutant SOD1 and VDAC has been recently challenged (Tan et al., 2013). According these observations, collected *in vitro* and *in vivo*, VDAC dysfunction is the result of a toxic association between mutant SOD1 and Bcl-2. Both SOD1 and VDAC compete for Bcl-2, which acts as a bridge between the two proteins. The conformational change in Bcl-2, induced by mutant SOD1 binding, exposes the toxic BH3 domain and favors the association of modified Bcl-2 with VDAC, resulting in a pathological alteration of OMM polarity and permeability, due to VDAC closure. This stream of mismatch associations seems to be a key event in disease initiation and progression. Interestingly, the administration of small peptides designed to target SOD1 and blocking its interaction with Bcl-2 might counteract these pathological alterations (Tan et al., 2013).

Collectively, the consequence of mutant SOD1 deposition onto OMM are: (i) conformational change of Bcl-2 preventing its anti-apoptotic association with Bax; (ii) impairment of VDAC1 function engaged by direct (SOD1-mediated) or indirect (Bcl-2 mediated) abnormal interactions with mutant SOD1, leading to changes in mitochondrial potential, morphology, protein composition and transport across membranes.

The specificity of mutant SOD1 in OMM targeting seems to be dictated by factors contained in the cytosol of the considered cell rather than its mitochondria. From these considerations, two hypotheses raised: it may exist a factor in spinal motor neurons that favors mislocalization, or, alternatively, there is a factor in the cytosol of other cells preventing it (Israelson et al., 2015). In a very recent publication, Cleveland investigated different heat labile factors that can block SOD1 mitochondria accumulation, selecting MIF as the most promising candidate. *MIF* (22q11.23) encodes for a small (12.5 kDa) protein, displaying multimeric and monomeric forms, with various functions: folding ATP-indipendent chaperone, thiol-oxidoreductase, and secreted cytokine.

MIF interacts with mutant SOD1 by preventing its binding with OMM proteins and the accumulation of misfolded SOD1. Other chaperones (Hsp70, Hsc70, aB-crystallin, cyclophilin-A, glutathione peroxidase) had no effect in blocking SOD1 misplacing onto mitochondria outer membrane (Israelson et al., 2015). The inhibitory activity of MIF is preserved even in mutant lacking thiol-oxydoreductase activity. This is an important consideration since misfolding and mislocalization are supposed to be driven by the lack of C57-C146 bond (Vehviläinen et al., 2014) and by mitochondrial binding induced by C111 (Ferri et al., 2006), respectively.

At onset, SOD1 G93A mice showed reduced MIF levels in motor neurons as an effect of rapid protein clearance (given the demonstration of conserved MIF transcription and active translation): the restoration of MIF to normal levels enhanced motor neurons survival. Since reducing misfolded SOD1 aggregates does not halt disease progression, *in vivo* toxicity seems to be driven by soluble misfolded SOD1, which can be reduced by the increase of MIF in a dose-dependent manner (Israelson et al., 2015).

The results presented about the toxic deposition of misfolded SOD1 onto OMM and those coming from the identification of MIF as the soluble factor responsible for the selective neutralization of misfolded SOD1 are intriguing. In particular, the misfolded accumulation of mutant SOD1 seems to be conserved in both active and inactive mutants, the latter presenting greater amount of misfolded proteins. This finding nicely fits with a general observation about the more aggressive phenotype (earlier onset, faster progression) associated with inactivating mutations (Sato et al., 2005). At the same time, even small amount of mutant misfolded proteins might produce a clinical phenotype in human while high levels of expressions of mutant constructs (different according the mutations considered) are required to show a neurologic deficit in mice.

More importantly, these studies provide novel clues about the selectivity of spinal cord involvement and disease progression. These results were confirmed and refined in independent experiments, although the same authors disclosed misfolded SOD1 in mitochondrial fractions isolated from patients’ lymphoblasts, challenging the cell type selectivity (Pickles et al., 2013). The identification of MIF as a candidate for ALS therapy is an intriguing discovery but this story is at its dawn. More confirmations are required to address unresolved issues including investigations on the degradative or secretive pathways acting in motor neurons to deprive intracellular MIF levels.

## Conclusion

Several groups demonstrated positive correlation between mutant SOD1 and ALS and the main pathogenetic hypothesis is a toxic gain of function. What is the toxic function gained and why it selectively strikes spinal motor neurons is still unexplained, but certainly not unexplored. One of the most investigated pathogenetic hypothesis underlining SOD1-related ALS focuses on mitochondrial toxicity of mutant SOD1. Fifteen years after the first observation on the co-localization of mutant SOD1 with vacuolated mitochondria in G93A mice spinal cord, the issue of SOD1 localization generated contradictory results but also promising answers. The mitochondrial localization of wild type and mutant SOD1 is a result definitively acquired and the hypothesis that the mutant function associated with ALS mutations involves the aberrant processing of superoxide anions is considered unlikely. Two major models have been advanced for mutant SOD1 accumulation inside mitochondria: increased content of mutant molecule, escaping physiological regulation inside IMS and selective deposition of misfolded protein onto OMM. These two routes are not mutually exclusive and studies supporting one model have also acknowledged the other as possible. The issue of submitochondrial localization hidden more relevant questions: which are the factors involved in SOD1 targeting towards mitochondria? Is the toxicity of mutant protein linked with aggregates (cytosolic or IMS), or soluble forms (SOD1 neutralized by MIF inside cytosol)? Are the mitochondria central for downstream ALS pathogenetic events or mitochondrial dysfunction adds to other intracellular mechanisms leading to motor neuron loss? Despite its key role in metabolism, mitochondria are involved in other fundamental cellular pathways, including calcium handling. Neuroblastoma cells expressing SOD1 mutants show higher levels of intracellular calcium compared to controls and are more vulnerable to further increase of calcium concentration (Goos et al., 2007; Jaiswal et al., 2009). These findings are supported by similar observations in G93A transgenic mice (Jaiswal and Keller, 2009). The reduction in calcium uptake does not seem the consequence of disturbed mitochondrial membrane potential. Conversely, it could be related to a differential expression of transporters and regulatory proteins involved in calcium handling, an hypothesis proposed to explain the selective vulnerability of spinal cord motor neurons to calcium (Fuchs et al., 2013).

Novel *in vivo* and *in vitro* experiments are required to investigate whether these mechanisms are directly influenced by altered IMS composition, primed by mutant SOD1 accumulation, or by impaired mitochondrial transport resulting from aberrant deposition of mutant SOD1 onto OMM.

In parallel, translational studies are mandatory to challenge the therapeutic potential of the targets observed to influence SOD1 misplacing.

## Conflict of Interest Statement

The authors declare that the research was conducted in the absence of any commercial or financial relationships that could be construed as a potential conflict of interest.
